# Flotation Restricted Environmental Stimulation Therapy for Chronic Pain: A Randomized Clinical Trial

**DOI:** 10.1001/jamanetworkopen.2021.9627

**Published:** 2021-05-14

**Authors:** Leonie F. Loose, Jorge Manuel, Matthias Karst, Laura K. Schmidt, Florian Beissner

**Affiliations:** 1 Somatosensory and Autonomic Therapy Research, Institute for Neuroradiology, Hannover Medical School, Hannover, Germany; 2 Institute of Aerospace Medicine, German Aerospace Center, Cologne, Germany; 3 Department of Anesthesiology and Intensive Care Medicine, Pain Clinic, Hannover Medical School, Hannover, Germany; 4 Insula Institute for Integrative Therapy Research, Hannover, Germany

## Abstract

**Importance:**

Flotation restricted environmental stimulation therapy (REST) is an emerging therapeutic intervention that, to our knowledge, has never been directly compared with an indistinguishable placebo in patients with chronic pain.

**Objective:**

To determine whether 5 flotation-REST sessions alleviate chronic pain.

**Design, Setting, and Participants:**

This single-blind, randomized clinical trial compared flotation-REST as an intervention for the treatment of chronic pain with indistinguishable placebo and wait-list control conditions at Hannover Medical School, Hannover, Germany. Men and women aged 18 to 75 years who had been diagnosed with chronic pain disorder with psychological and somatic factors (*International Statistical Classification of Diseases and Related Health Problems, 10th Revision, German Modification*, code F45.41) by physicians at the study center were randomly assigned to 1 of the 3 groups. Data were collected from June 26, 2018, to June 18, 2020.

**Interventions:**

Patients in the intervention and placebo groups underwent 5 treatment sessions lasting 60 to 90 minutes, each session separated by 4 days. The placebo treatment was delivered in the same floating tank but controlled for effortless floating and environmental stimulus restriction. Patients in the wait-list control group did not receive any additional treatment but were asked to continue any ongoing treatments at the time of enrollment.

**Main Outcomes and Measures:**

The primary outcome was a change in pain intensity 1 week after the last treatment session. The assessment was repeated at 12 and 24 weeks. Secondary outcomes included pain-related disability, pain area, pain widespreadness (number of body regions affected by pain), anxiety, depression, and quality of life, as well as several other short-term outcomes.

**Results:**

A total of 99 patients (mean [SD] age, 51.7 [12.3] years; 80 women [81%]) were included in the study. No differences were found among the groups in the primary outcomes (mean [SD] change in maximum pain: −7.6 [19.7] for the intervention group, −5.8 [12.7] for the placebo group, and 0.4 [14.0] for the wait-list control group; mean [SD] change in mean pain: −2.1 [19.4] for the intervention group,−4.2 [16.2] for the placebo group, and 2.0 [12.6] for the wait-list control group). Long-term secondary outcomes did not show significant differences. In the short term, patients in the intervention group showed significant improvements in pain intensity (−17.0 [17.1]; *P* < .001), relaxation (23.9 [22.6]; *P* < .001), anxiety (−10.1 [8.4]; *P* < .001), pain area (−3.6% [7.4%]; *P* < .001), and widespreadness (−2.0 [3.0]; *P* < .001), and similar changes where observed in the placebo group.

**Conclusions and Relevance:**

Patients with chronic pain experienced no long-term benefits from the 5 flotation-REST interventions. Clinically relevant short-term changes in pain in the placebo group suggest that improvements may not be caused by environmental stimulus restriction or effortless floating as previously thought.

**Trial Registration:**

ClinicalTrials.gov Identifier: NCT03584750

## Key Points

**Question:**

Do 5 sessions of flotation restricted environmental stimulation therapy alleviate chronic pain?

**Findings:**

This randomized clinical trial of 99 participants included intervention, placebo, and wait-list control groups. No differences among the groups were found for the primary end point (change in pain intensity 1 week after the last treatment session) or for secondary long-term outcomes; in the short term, a significant increase in relaxation and decrease in anxiety, pain intensity, pain area, and widespreadness (number of body regions affected by pain) were observed.

**Meaning:**

Patients with chronic pain experienced no long-term benefits from the 5 treatment sessions.

## Teaser

This randomized clinical trial assesses whether 5 sessions of flotation–restricted environmental stimulation therapy alleviate chronic pain in adults.

## Introduction

Chronic pain—that is, pain lasting for more than 3 months—is a major health problem. Approximately 1 in 5 adults experiences chronic pain with subsequent impairment in life and work activities.^1^ Furthermore, pain is one of the essential factors associated with sick leave, use of health care services, and unemployment.^2^ Remarkably, despite the variety of therapeutic options (eg, pharmacological therapy, physiotherapy, and psychotherapy), many patients are unsatisfied with their treatment,^3,4,5,6^ demonstrating the need for treatment alternatives.

Mind-body therapies, such as yoga, relaxation techniques, and mindfulness-based approaches, can be a helpful adjunct to treat chronic pain.^7^ Both somatic and psychological factors contribute to the development, maintenance, and exacerbation of chronic pain. Permanent psychosocial stress leads to complex neural, hormonal, and behavioral changes that facilitate the development of chronic pain.^8^ At the same time, chronic pain itself induces a continuous stress reaction.^9,10^ Mind-body therapies aim at breaking the vicious circle by reducing muscle tension, cognitive load, arousal, and anxiety by eliciting a relaxation response.^7,11^

Flotation restricted environmental stimulation therapy (REST), among other effects, has been associated with producing a relaxation response.^12^ Patients lie in a tank, floating effortlessly on water with a high salt concentration. The water is heated to skin temperature, and the tank is shielded against light and sound. As a consequence, mechanical, thermal, visual, and acoustic stimuli are reduced to a minimum. Previous studies have shown positive effects of flotation-REST, such as increased relaxation,^13^ lowered stress levels,^14,15^ improved sleep quality,^16^ reduced anxiety,^14,15,17,18^ and pain relief.^15,19^

These studies, however, differed in design, sample size, inclusion criteria, and dosage of flotation-REST, which hinders their comparison. Several studies have examined the effects of flotation-REST on chronic pain,^14,15,16,19,20^ but the effects on chronic pain lasting more than 72 hours were only assessed once.^20^ The most significant limitation of these studies, however, is the lack of an indistinguishable placebo control needed for patient-blind controlled studies.^17^ So far, flotation-REST was mostly compared with either a wait-list control condition,^21,22^ sitting in an armchair,^20^ lying in a soundproof cubicle,^23^ or so-called dry floating,^24^ all of which are distinguishable from the actual intervention.

Herein, we introduce an indistinguishable placebo intervention for comparison with flotation-REST. Because it has been suggested that (1) environmental stimulus restriction resulting from the tank environment and (2) muscle relaxation and reduction of proprioceptive input resulting from effortless floating are the essential factors for inducing a relaxation response,^25,26^ we aimed at reducing these factors to a credible minimum while leaving all other aspects unchanged.

We applied our new placebo condition to investigate whether patients with chronic pain would benefit from 5 consecutive sessions of flotation-REST. Our hypothesis was that the intervention group would show larger improvements than the placebo group. We investigated both short- and long-term effects and were particularly interested in pain relief 1 week after the intervention.

## Methods

### Study Participants

The study took place at Hannover Medical School, Hannover, Germany. The study protocol complied with the Declaration of Helsinki,^27^ was approved by the local ethics committee, and was registered at ClinicalTrials.gov; the trial protocol and statistical analysis plan are found in Supplement 1. All patients provided written informed consent. This report follows the Consolidated Standards of Reporting Trials (CONSORT) reporting guideline for randomized studies.^28^ Unless otherwise indicated, data are expressed as mean (SD).

We enrolled men and women aged 18 to 75 years who had been diagnosed with chronic pain disorder with psychological and somatic factors (*International Statistical Classification of Diseases and Related Health Problems, 10th Revision, German Modification* [*ICD-10-GM*], code F45.41 [equivalent to *Diagnostic and Statistical Manual of Mental Disorders* (Fourth Edition) code 307.89]), who had no prior experience with flotation-REST. Exclusion criteria were pregnancy, acute illness, contagious disease, acute major depression, epilepsy, claustrophobia, schizophrenia, incontinence, and suspected literacy and cognitive barriers to understanding the instructions. Patients were recruited at the pain outpatient department and the clinic for rehabilitation medicine at Hannover Medical School. The diagnosis was made by pain specialists, and exclusion criteria were checked anamnestically.

### Study Design

To investigate the effects of flotation-REST on chronic pain, we randomly assigned participants to 1 of 3 groups using a central telephone randomization procedure prepared by an investigator not involved in patient care (F.B.). The blocked randomization list was created with a seed-based pseudo–number generator.

Participants were informed that they would receive 1 of 2 different kinds of flotation-REST (without further specification) or serve as wait-list controls. The intervention group received a regular intervention with effortless floating and normal environmental stimulus restriction. The placebo group received a modified intervention in the same tank (see below). Participants in both groups underwent 5 treatment sessions. This number was based on feasibility. Each of the sessions lasted 60 to 90 minutes. After 60 minutes, participants were contacted via intercom, and they could decide to end the session or to continue for another 30 minutes. The wait-list control group did not receive any additional treatment. Patients in this group received 1 session as compensation after complete data collection. All 3 groups were asked to continue their prescribed treatments and not to start any new intervention during the study.

### Flotation-REST Intervention

Treatment sessions took place in a commercially available flotation pod (Tranquility; Floataway). Its water was constantly kept at skin temperature (mean, 35.0 °C [0.2 °C]). A high buoyancy was achieved by increasing water density to a mean of 1.27 (0.01) kg/L by adding nontoxic magnesium sulfate (Epsom salt). Participants wore swimwear and sound-insulating wax earplugs. The tank was installed in a quiet, darkened room. An intercom system enabled communication with the staff during the measurements. Patients were encouraged to switch off the lights inside the tank, which was not further controlled. Thirteen participants, 6 in the intervention group and 7 in the placebo group, requested to float with an open lid. One participant did so during 2 sessions, another during 4 sessions, and all others during each session. Just 1 participant (in the placebo group) requested to float with a completely opened lid. All others opened the lid less than 5 cm.

Participants were asked to refrain from shaving and drinking coffee 6 hours before each session. The consumption of alcohol was not allowed 24 hours before the session. Before each session, participants were asked whether they had followed this protocol and whether they had any acute illness.

### Placebo Intervention

Our placebo intervention was designed to mimic regular flotation-REST as much as possible but with strongly reduced levels of environmental stimulus restriction and effortless floating. Sessions took place in the same tank.

We reduced buoyancy by decreasing the mean water density to 1.10 (0.05) kg/L (freshwater density, approximately 1.00 kg/L). Water depth was adjusted to a mean of 16.2 (0.8) cm compared with 25.2 cm in the intervention group. These 2 changes ensured that participants rested on the bottom of the tank. Participants were told that the low water level was necessary for safety reasons.

Environmental stimulus restriction was reduced by applying visual, acoustic, thermal, and tactile stimulation. The interior lighting of the tank stayed on throughout the session, and background music (a playlist of acoustic guitar music without vocals) was played constantly. Patients wore soft earplugs compared with the more insulating wax earplugs used by the intervention group. Because participants rested on the floor, they felt the warmth from the tank’s underfloor heating on their backs. Furthermore, a blood pressure cuff was attached to the lower leg and inflated at random intervals (mean, 4.0 [0.7] minutes). Finally, patients were asked to rate their current pain and relaxation levels a mean of every 10.3 (2.4) minutes (eTable 1 in Supplement 2). The goal was to prevent deep relaxation by changing stimuli and active cognitive processes. Treatment credibility and expectancy were assessed using the 4-item scale of Borkovek and Nau.^29^

We clustered sessions of the same group together, because changing the salt concentration after every session would have been unfeasible. Hence, both groups floated at different times of the year (eTable 2 in Supplement 2). Appointments were made directly after randomization for the scheduled time in the year.

### Outcome Measures and Follow-up

The predefined primary outcome was a change in pain intensity (maximum and mean) assessed retrospectively for 1 week using a validated 101-point numerical rating scale (NRS) ranging from 0 (no pain) to 100 (worst imaginable pain). The NRS has been shown to be a simple and reliable tool for assessing pain changes in patients with chronic pain.^30,31,32^ This and all secondary long-term outcomes were collected at baseline and in 3 follow-ups (1, 12, and 24 weeks after the last intervention and 4, 15, and 27 weeks after baseline for the wait-list control group).

Predefined secondary long-term outcomes comprised pain-related disability (Pain Disability Index [range, 0-70, with higher scores indicating greater disability]^33^), pain area, pain widespreadness (number of body regions affected by pain, as assessed by the Widespread Pain Index [WPI; range, 0-19, with higher scores indicating more body regions affected by pain]^34^), trait anxiety (State-Trait Anxiety Inventory [range, 20-80, with higher scores indicating greater anxiety]^35^), depression (Beck Depression Inventory-II [range, 0-63, with higher scores indicating greater severity of depression]^36^), quality of life (12-Item Short Form Health Survey [mean *t* score, 50 [10], with higher scores indicating better physical and mental health]^37^), sleep quality (NRS from 0 [not impaired by pain] to 100 [very much impaired] retrospectively for 1 week), and use of pain medication. To analyze the use of pain medication, we defined medication steps that corresponded to the most potent pain medication taken by each patient, where 0 indicates no pain medication; 1, nonopioid analgesics; 2, analgesic adjuvants; 3, cannabinoids; 4, weak opioids; and 5, strong opioids. Adjustment of dosage or switch of drugs within a group were not considered.

All predefined secondary short-term outcomes were assessed before and after each session, concretely before the shower that preceded the session and then after the shower that followed the session. These outcomes comprised current pain intensity (NRS as above) and level of relaxation (NRS from 0 [not relaxed at all] to 100 [maximally relaxed]), pain area, WPI, state anxiety (State-Trait Anxiety Inventory^35^), heart rate variability (see below), and unusual bodily sensations during the intervention (patient report).

Pain area and WPI were derived from electronic pain drawings acquired with the SymptomMapper application.^38^ The WPI has been used for the diagnosis of fibromyalgia^34,39^ and has been associated with central sensitization.^40^

Heart rate and heart rate variability were derived from electrocardiographic recordings of 300 beats recorded from the participants’ wrists using a commercially available system (Medi/DINAMIKA; Nilas MV GmbH). Participants were seated and instructed not to talk during the measurement. The recordings were analyzed with Nilas Medi software, version 5.2.8 (Nilas MV GmbH), using automated peak detection and artifact rejection. Measures included heart rate, high-frequency (0.15-0.4 Hz) and low-frequency (0.04-0.15 Hz) power spectral density, their quotient (low divided by high frequency), SD of normal-to-normal R-R intervals (SDNN), root-mean-square of successive differences (RMSSD), coefficient of variation, and proportion of pairs of successive normal beats that differed by more than 50 milliseconds. Heart rate variability measures provided information about cardiac regulation by the autonomic nervous system.^41^

### Statistical Analysis

A previous exploratory study^15^ showed significant differences between flotation-REST (n = 20) and no treatment (n = 17) in its effect on muscle tension pain. From the reported data of Kjellgren et al,^15^ we estimated a Cohen *d* of 0.83 yielding a required sample size of 24 participants per group for a 2-tailed, 2-sample *t* test (α = .05; 1 − β = .95; allocation ratio, 1:1). Hence, we aimed for 25 participants with complete data per group.

Statistical analyses included 1-way analyses of variance for long-term outcomes, χ^2^ tests for dichotomous outcomes, and 2-tailed Welch *t* tests for comparing 2 groups. We also computed individual 2-tailed paired *t* tests for short-term outcomes. To correct for potential type I errors due to the multiple secondary outcomes, we applied a Bonferroni correction. We report corrected *P* values, with *P* < .05 considered statistically significant. All analyses were performed in Python, version 3.5.2, using NumPy, version 1.17.4, and SciPy, version 0.19.1 (Python Software Foundation). We included all available data in our analyses (pairwise deletion) because we could not find any systematic reason for dropping out of the study (eTable 3 in Supplement 2).

## Results

**Figure 1.**
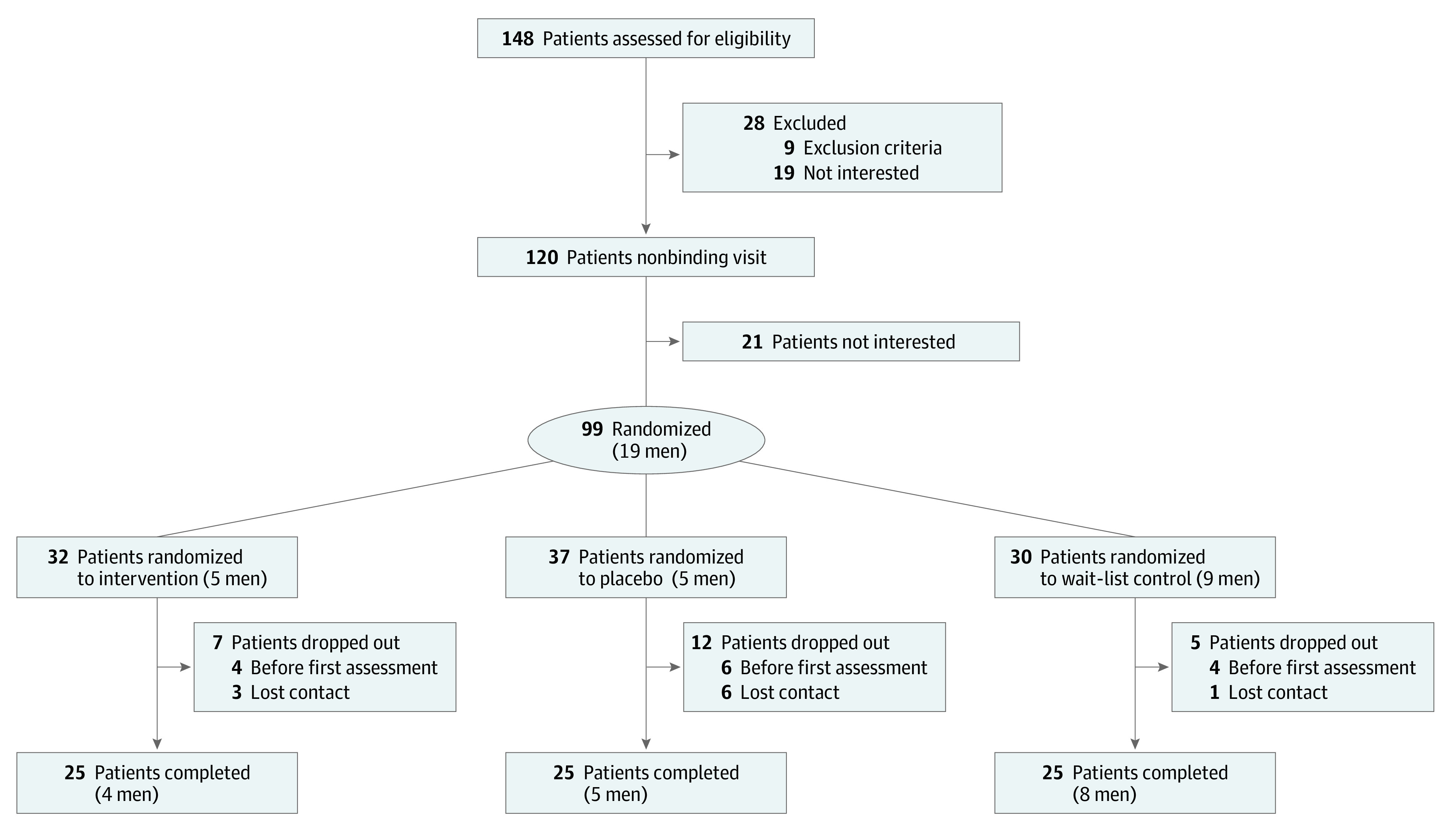
Study Flowchart Dropout reasons are listed in eTable 3 in Supplement 2.

We recruited 99 participants (mean age, 51.7 [12.3] years; 80 women [81%] and 19 men [19%]) after screening 148 patients from June 26, 2018, to June 18, 2020. Fourteen of the participants dropped out before any measurement, and 10 did not complete any of the follow-up assessments (Figure 1 and eTable 3 in Supplement 2). Baseline characteristics (Table 1) were well balanced among the 3 groups. Participants reported moderate to severe pain on the NRS (maximum, 73.9 [16.3]; mean, 50.1 [17.3]) and had moderate levels of depression (Beck Depression Inventory II score, 22.3 [12.5]) and trait anxiety (State-Trait Anxiety Inventory part X2 score, 52.3 [11.9]). The sex distribution was representative of the patients with *ICD-10-GM* code F45.41 in the pain outpatient department of Hannover Medical School (74.3% female).

**Table 1.** Baseline Characteristics of the Study Participants

Measure	Study group^a^		
	Intervention (n = 32)	Placebo (n = 37)	Wait-list control (n = 30)
Sex, No. (%)			
Women	27 (84)	32 (86)	21 (70)
Men	5 (16)	5 (14)	9 (30)
Age, y	52.8 (11.5)	48.8 (13.8)	54.2 (10.1)
Medication step, No. (%)^b^			
0: No pain medication	2 (6)	5 (14)	2 (7)
1: Nonopioid analgesics	6 (19)	6 (16)	3 (10)
2: Analgesic adjuvants	14 (44)	9 (24)	11 (37)
3: Cannabinoids	0	0	0
4: Weak opioids	4 (13)	7 (19)	2 (7)
5: Strong opioids	2 (6)	4 (11)	8 (27)
Mean medication step^b^	2.1 (1.3)	2.3 (1.7)	2.8 (1.7)
Pain outcome, NRS score^c^			
Maximum pain (last week)	71.1 (16.8)	75.4 (15.4)	75.1 (16.0)
Mean pain (last week)	49.7 (16.5)	49.1 (17.7)	52.1 (17.0)
Pain Disability Index^d^	34.3 (13.9)	34.3 (13.3)	38.4 (16.5)
State-Trait Anxiety Inventory score^e^	52.5 (10.8)	50.2 (11.9)	54.6 (12.6)
Beck Depression Inventory-II score^f^	23.3 (11.9)	20.4 (13.0)	23.5 (12.3)
Sleep impairment NRS score^c^	46.2 (31.1)	61.1 (28.7)	66.7 (27.5)
SF-12^g^			
Physical component	29.5 (6.8)	31.5 (8.7)	28.7 (6.7)
Mental component	38.3 (11.2)	39.4 (12.4)	39.2 (14.5)

Abbreviations: NRS, numerical rating scale; SF-12, 12-Item Short Form Health Survey.

^a^ Unless otherwise indicated, data are expressed as mean (SD).

^b^ Corresponds to the most potent pain medication taken. Four participants in the intervention group, 6 in the placebo group, and 4 in the wait-list control group dropped out before any measurement.

^c^ Scores range from 0 to 100, with higher scores indicating maximum pain/impairment.

^d^ Scores range from 0 to 70, with higher scores indicating greater disability.

^e^ Scores range from 20 to 80, with higher scores indicating greater disability.

^f^ Scores range from 0 to 63, with higher scores indicating greater severity of depression.

^g^ Mean (SD) *t* score, 50 (10), with higher scores indicating better physical and mental health.

We did not observe significant differences in credibility or expectancy between the intervention and placebo groups (Table 2). No participant expressed any doubts concerning the credibility of the received intervention, nor did any participant indicate that the placebo intervention was not flotation-REST.

**Table 2.** Long-term Effects of Intervention

Measure	Study group^a^			Statistic
	Intervention	Placebo	Wait-list control	
**Change from baseline at 1 wk**				
Maximum pain, NRS score^b^	−7.6 (19.7)	−5.8 (12.7)	0.4 (14.0)	F_2,73_ = 1.7
Mean pain, NRS score^b^	−2.1 (19.4)	−4.2 (16.2)	2.0 (12.6)	F_2,73_ = 0.9
30% Reduction in maximum pain, No. (%)	5 (20)	4 (16)	2 (8)	χ^2^ = 1.3
30% Reduction in mean pain, No. (%)	5 (20)	5 (20)	1 (4)	χ^2^ = 2.9
Pain Disability Index^c^	−1.7 (10.7)	−4.2 (8.4)	0.0 (5.1)	F_2,72_ = 1.5
State-Trait Anxiety Inventory score^d^	−1.7 (5.9)	−1.3 (5.6)	−1.8 (4.3)	F_2,72_ = 0.1
Beck Depression Inventory-II score^e^	−3.3 (10.8)	−2.0 (4.7)	−2.2 (4.8)	F_2,72_ = 0.2
SF-12 score^f^				
Physical component	3.0 (5.9)	2.3 (8.3)	0.7 (4.5)	F_2,72_ = 0.8
Mental component	2.9 (8.1)	2.4 (6.1)	−0.7 (5.8)	F_2,72_ = 1.9
Sleep impairment, NRS score^b^	−6.8 (27.2)	−17.4 (25.4)	−7.4 (15.6)	F_2,73_ = 1.6
Medication step^g^	−0.3 (0.8)	−0.0 (0.7)	−0.4 (1.1)	F_2,72_ = 1.1
Pain area, %	−5.0 (14.7)	−3.1 (7.7)	NA	*t*_39_ = −0.5
Widespread Pain Index^h^	−3.1 (3.7)	−0.6 (3.5)	NA	*t*_39_ = −2.2
Credibility and expectancy^i^	1.2 (2.3)	0.9 (3.5)	NA	*t*_48_ = 0.3
**Change from baseline at 12 wk**				
Maximum pain, NRS score^b^	−3.6 (15.7)	−3.4 (14.7)	−2.5 (14.3)	F_2,72_ = 0.0
Mean pain, NRS score^b^	−2.0 (19.6)	−1.8 (11.6)	−1.0 (13.8)	F_2,72_ = 0.0
30% Reduction in maximum pain, No. (%)	2 (8)	2 (8)	2 (8)	χ^2^ = 0.0
30% Reduction in mean pain, No. (%)	8 (32)	4 (16)	3 (12)	χ^2^ = 2.8
Pain Disability Index^c^	−4.8 (11.6)	−0.5 (8.9)	−0.2 (9.9)	F_2,72_ = 1.5
State-Trait Anxiety Inventory score^d^	−3.0 (6.4)	0.0 (6.3)	−3.2 (3.5)	F_2,72_ = 2.5
Beck Depression Inventory-II score^e^	−3.6 (9.0)	−0.5 (6.2)	−2.2 (4.4)	F_2,72_ = 1.2
SF-12 score^f^				
Physical component	3.0 (7.8)	0.7 (6.0)	0.9 (5.0)	F_2,72_ = 1.0
Mental component	−0.4 (8.9)	1.4 (7.7)	−1.0 (5.5)	F_2,72_ = 0.6
Sleep impairment NRS score^b^	1.6 (25.0)	−7.6 (22.6)	−8.4 (15.5)	F_2,72_ = 1.6
Medication step^g^	0.2 (1.0)	−0.2 (0.8)	−0.4 (1.1)	F_2,72_ = 2.4
**Change from baseline at 24 wk**				
Maximum pain, NRS score^b^	−2.4 (19.6)	−0.8 (15.3)	−2.1 (14.3)	F_2,73_ = 0.1
Mean pain, NRS score^b^	−0.2 (13.7)	−2.4 (14.1)	−2.4 (13.4)	F_2,73_ = 0.2
30% Reduction in maximum pain, No. (%)	3 (12)	3 (12)	2 (8)	χ^2^ = 0.2
30% Reduction in mean pain, No. (%)	4 (16)	5 (20)	5 (20)	χ^2^ = 0.1
Pain Disability Index^c^	0.2 (12.0)	−3.7 (11.2)	0.9 (12.9)	F_2,73_ = 1.0
State-Trait Anxiety Inventory score^d^	−1.9 (5.9)	−0.8 (6.7)	−0.7 (4.8)	F_2,73_ = 0.3
Beck Depression Inventory-II score^e^	−0.8 (7.2)	−0.9 (7.0)	−2.0 (6.5)	F_2,73_ = 0.2
SF-12 score^f^				
Physical component	2.7 (7.0)	3.2 (8.2)	−0.1 (5.2)	F_2,73_ = 1.5
Mental component	0.1 (5.9)	−1.0 (8.2)	−0.6 (6.2)	F_2,73_ = 0.2
Sleep impairment NRS score^b^	2.6 (20.8)	−15.4 (28.3)	−8.0 (17.1)	F_2,73_ = 3.9
Medication step^g^	0.0 (1.2)	0.0 (0.7)	−0.5 (1.4)	F_2,73_ = 1.7

Abbreviations: NA, not applicable; NRS, numerical rating scale; SF-12, 12-Item Short Form Health Survey.

^a^ Unless otherwise indicated, data are expressed as mean (SD). Maximum and mean pain NRS scores are primary outcomes.

^b^ Scores range from 0 to 100, with higher scores indicating maximum pain/impairment.

^c^ Scores range from 0 to 70, with higher scores indicating greater disability.

^d^ Scores range from 20 to 80, with higher scores indicating greater anxiety.

^e^ Scores range from 0 to 63, with higher scores indicating greater severity of depression.

^f^ Mean (SD) *t* score, 50 (10), with higher scores indicating better physical and mental health.

^g^ Ranges from 0 to 5, corresponding to most potent pain medication used. Steps are described in Table 1.

^h^ Scores range from 0 to 19, with higher scores indicating more body regions affected by pain.

^i^ Scores range from 0 to 12, with higher scores indicating higher credibility and expectancy in the treatment.

**Figure 2.**
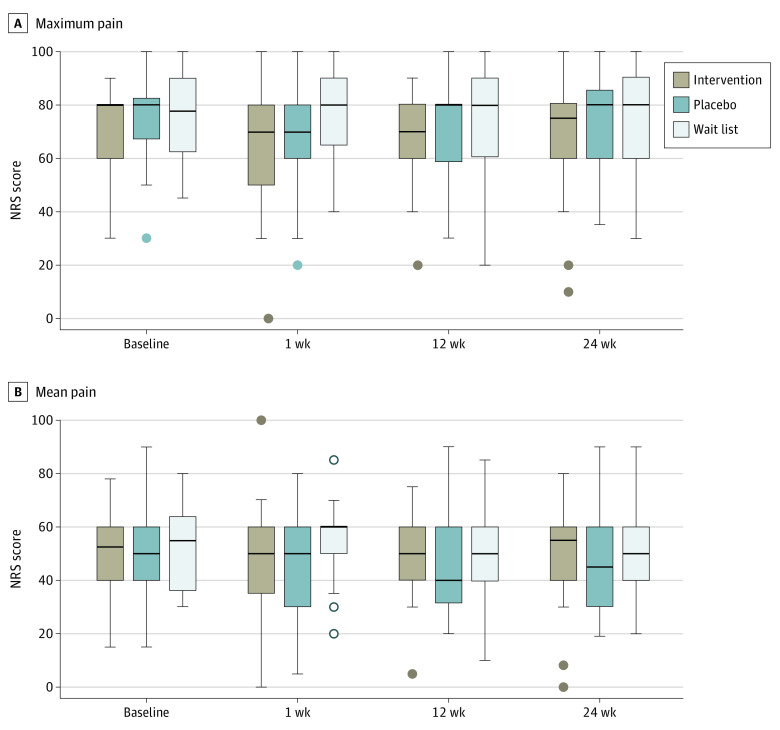
Primary Outcomes at Baseline and 1, 12, and 24 Weeks After the End of the Intervention NRS indicates numerical rating scale.

Table 2 shows changes from baseline to 1, 12, and 24 weeks after the end of the intervention. At no point did we observe any significant differences among the primary outcomes (change in maximum pain: −7.6 [19.7] in the intervention group, −5.8 [12.7] in the placebo group, and 0.4 [14.0] in the wait-list control group; change in mean pain: −2.1 [19.4] in the intervention group, −4.2 [16.2] in the placebo group, and 2.0 [12.6] in the wait-list control group) (Figure 2). There were also no differences in secondary long-term outcomes among the 3 groups.

Table 3 shows changes of the secondary short-term outcomes comparing the preintervention and postintervention periods. Patients in the intervention group showed significant improvements in pain intensity (−17.0 [17.1]; *P* < .001), relaxation (23.9 [22.6]; *P* < .001), anxiety (−10.1 [8.4]; *P* < .001), pain area (−3.6% [7.4%]; *P* < .001), and WPI (−2.0 [3.0]; *P* < .001). Effect sizes ranged from medium to large. Furthermore, small- to medium-sized effects were observed for heart rate (−2.4 [5.7] bpm; *P* < .001) and heart rate variability parameters SDNN (3.7 [9.8] milliseconds; *P* < .001), RMSSD (1.5 [4.8] milliseconds; *P* = .02), and coefficient of variation (0.4% [1.2%]; *P* = .01). Similar changes, however, were also observed in the placebo group. Significant differences between the 2 groups were found for relaxation, with more extensive improvements in the placebo group (34.4 [26.3]; *P* = .008). We did not observe differences between the sessions, nor did we observe any cumulative effects (eFigure in Supplement 2). Thirty-four participants (18 of 28 [64%] remaining in the intervention group and 16 of 31 [52%] remaining in the placebo group) experienced unusual bodily sensations during their sessions, ranging from tingling and heaviness to out-of-body experiences (eTable 4 in Supplement 2).

**Table 3.** Short-term Effects of the Intervention

Measure	Study group^a^				Statistical analysis		
	Intervention		Placebo		∆Intervention vs ∆Placebo	Preintervention vs postintervention	Effect size, Cohen *d*
	Preintervention	Postintervention	Preintervention	Postintervention			
Pain intensity^b^	51.3 (20.6)	34.3 (20.4)	46.0 (21.3)	22.7 (18.4)	*t*_263_ = 2.8	Intervention *t*_128_ = −11.2^c^	−0.99
						Placebo *t*_135_ = −14.1^c^	−1.21
Relaxation^b^	47.2 (24.2)	71.1 (22.5)	46.9 (22.5)	81.3 (17.9)	*t*_263_ = −3.5^d^	Intervention *t*_128_ = 12.0^c^	1.06
						Placebo *t*_135_ = 15.2^c^	1.31
Pain area, %	10.6 (12.9)	7.0 (11.2)	7.8 (9.2)	4.4 (7.3)	*t*_263_ = −0.3	Intervention *t*_128_ = −5.5^c^	−0.49
						Placebo *t*_135_ = −6.7^c^	−0.58
WPI^e^	6.6 (4.5)	4.6 (4.2)	5.4 (4.1)	3.4 (3.7)	*t*_263_ = 0.1	Intervention *t*_128_ = −7.6^c^	−0.67
						Placebo *t*_135_ = −7.9^c^	−0.68
State-Trait Anxiety Inventory score^f^	45.0 (12.0)	34.8 (9.9)	44.6 (11.0)	32.3 (8.4)	*t*_263_ = 1.9	Intervention *t*_128_ = −13.6^c^	−1.21
						Placebo *t*_135_ = −14.9^c^	−1.28
Heart rate, bpm	80.9 (9.8)	78.6 (9.7)	80.0 (12.0)	77.1 (12.3)	*t*_244_ = 0.6	Intervention *t*_116_ = −4.5^c^	−0.42
						Placebo *t*_128_ = −5.4^c^	−0.49
High-frequency power, ms^2^	74.7 (104.7)	85.1 (103.8)	99.3 (103.2)	126.0 (155.4)	*t*_244_ = −1.2	Intervention *t*_116_ = 1.8	NA
						Placebo *t*_128_ = 2.3	NA
Low-frequency power, ms^2^	300.9 (433.5)	351.0 (456.1)	360.8 (542.7)	433.5 (644.1)	*t*_244_ = −0.5	Intervention *t*_116_ = 1.6	NA
						Placebo *t*_128_ = 1.6	NA
Low-divided by high-frequency power	6.0 (6.1)	5.7 (5.4)	5.5 (5.9)	4.9 (4.0)	*t*_244_ = 0.4	Intervention *t*_116_ = −0.8	NA
						Placebo *t*_128_ = −1.4	NA
SDNN, ms	25.5 (13.2)	29.3 (14.0)	28.3 (12.4)	30.7 (15.2)	*t*_244_ = 0.9	Intervention *t*_116_ = 4.1^c^	0.38
						Placebo *t*_128_ = 2.5	NA
RMSSD, ms	13.4 (7.7)	14.8 (7.7)	16.7 (9.6)	17.6 (10.5)	*t*_244_ = 0.5	Intervention *t*_116_ = 3.3^g^	0.30
						Placebo *t*_128_ = 1.1	NA
Coefficient of variation, %	3.4 (1.6)	3.8 (1.7)	3.7 (1.6)	3.8 (1.8)	*t*_244_ = 1.4	Intervention *t*_116_ = 3.3^g^	0.31
						Placebo *t*_128_ = 1.4	NA
pNN50, %	1.2 (3.4)	1.5 (3.3)	3.0 (6.5)	3.3 (5.9)	*t*_244_ = 0.0	Intervention *t*_116_ = 1.5	NA
						Placebo *t*_128_ = 0.5	NA
Float duration, min	71.0 (12.4)		68.6 (11.7)		*t*_263_ = 1.6	NA	NA
Gap between floats, d	4.3 (2.4)		4.6 (2.5)		*t*_205_ = −1.0	NA	NA

Abbreviations: NA, not applicable; pNN50, proportion of pairs of successive normal beats that differed by more than 50 ms; RMSSD, root-mean-square of successive differences; SDNN, SD of normal-to-normal R-R intervals.

^a^ All outcomes were assessed before and after each flotation session, and mean results were calculated for 5 sessions, except for flotation duration and gap between flotation sessions.

^b^ Scores range from 0 to 100, with higher scores indicating maximum pain/relaxation.

^c^ *P* < .001.

^d^ *P* < .01.

^e^ Scores range from 0 to 19, with higher scores indicating more body regions affected by pain.

^f^ Scores range from 20 to 80, with higher scores indicating greater anxiety.

^g^ *P* < .05.

## Discussion

This randomized clinical trial showed that an intervention of 5 sessions of flotation-REST offers no significant long-term therapeutic benefits for patients who have been diagnosed with chronic pain disorder with somatic and psychological factors. The observed therapeutic effects were short lived and could no longer be detected 1 week after the intervention.

The absence of long-term effects of flotation-REST compared with the wait-list control condition differs from previous reports of sustained improvements in pain intensity,^15^ pain area,^16,20^ anxiety,^16,20,21^ sleep impairment,^16,20,21^ and depression.^15,16,20,21^ Some of these differences might be explained by the elapsed time until follow-up, which varied at 0 days,^15^ 3 days,^16^ 4 months,^20^ and 6 months.^21^ Furthermore, the designs of these studies incorporated higher numbers of sessions (9,^15^ 12,^20,21^ and 33^16^). More sessions might be required to achieve sustained therapeutic effects, but this needs to be addressed in future studies.

Special consideration should be given to the previous study data that we used to estimate required sample size.^15^ Pain ratings in the previous study seem to have been collected directly after the last intervention. Therefore, patients in the previous study may have experienced acute effects, given the short-term effects observed in our study (Table 3).

Another significant difference between previous studies and ours is the targeted disease. Previous studies included patients with “stress-related pain of muscle tension type,”^20^ “muscle tension pain,”^15^ and generalized anxiety disorder^21^; patients in our study had chronic pain disorder with somatic and psychological factors. This diagnosis describes chronic pain with a somatic origin, but with psychological factors that play an essential role for its severity, exacerbation, or maintenance. It is conceivable that flotation-REST is less suitable for patients with this diagnosis.

In the short term, the intervention group showed significant and often large improvements in pain intensity, pain area, WPI, and state anxiety. These findings are compatible with our current understanding of chronic pain, with mutual interdependencies among pain, stress, and cognitive factors. Furthermore, we observed a decrease in heart rate and an increase in heart rate variability (SDNN, RMSSD, and coefficient of variation), which points to an increase in parasympathetic tone, compatible with increased relaxation. Our observations are also in line with those of previous studies showing short-term effects in terms of pain relief,^15,19^ improved quality of sleep,^16^ lower stress levels,^14,15,16^ and reduction of anxiety.^14,15,16,17^ However, unexpectedly, our placebo group showed very similar effects to the intervention group.

To interpret these findings, we must critically review our placebo intervention. The specific effects we tried to control for were caused by effortless floating and restricted environmental stimulation, both of which are considered essential by many researchers in the field.^25,26^ We were able to strongly reduce both factors. However, the obtained short-term results make it highly unlikely that any of these factors play an important role in explaining the observed short-term effects on our patients. This finding is consistent with those of previous studies showing that the presence of light inside the floating tank has no effect on treatment outcome,^42^ and that even the importance of water immersion is limited.^24^ The latter is particularly interesting, because to our knowledge, this study is the only one in which the control condition took place in the same environment as the intervention (namely, inside a tank).

### Limitations

This study has some limitations, the main one being the heterogeneity of pain symptoms subsumed under *ICD-10-GM* code F45.41. Further differentiation of comorbidities, triggering somatic events, or pain localization was not performed, which could explain the variability in long-term assessments.

 Furthermore, seasonal effects resulting from our cluster approach may have influenced the results. Such effects have been observed in musculoskeletal pain due to increased physical activity in summer.^43^

In addition, the possible effects of the heated floor in the placebo group should be considered. Patients with fibromyalgia (often classified as *ICD-10-GM* code F45.41) showed sustained pain reduction as long as 6 months after whole-body heat therapy.^44^

Moreover, in contrast to studies in which participants floated while naked, participants in this study wore swimwear to avoid potential discomfort, because the dressing room was used by other patients. Hence, tactile stimuli could not be completely excluded.

Two additional factors that might have contributed to the therapeutic effect and that were not controlled in our study are expectation and attention.^45^ Our participants showed high expectations toward the treatment, which may have induced unspecific effects in all groups. Among these effects, expectation-induced analgesia is well researched and mediated by various endogenous neurotransmitters such as opioids, serotonin, and oxytocin.^46^

## Conclusions

To our knowledge, this is the first randomized, placebo-controlled clinical trial of flotation-REST in patients who have been diagnosed with chronic pain with somatic and psychological factors (*ICD-10-GM* code F45.41). Our results show that these patients experienced no long-term benefits from the 5 treatment sessions. Significant improvements occurred in the short term, but they do not seem to be caused by environmental stimulus restriction or effortless floating, as previously thought.

## Notes

Supplement 1. Trial Protocol and Statistical Analysis Plan

Supplement 2. **eFigure.** Short-term Differences (Postintervention Minus Preintervention) for Each Session **eTable 1.** Measurements During Placebo Floating **eTable 2.** Time of the Year When the Different Groups Floated **eTable 3.** Dropout Reasons **eTable 4.** Unusual Sensations During Flotation-REST and Placebo Floating **eTable 5.** Normalized Heart Rate Variability Measures

Supplement 3. Data Sharing Statement
