# Supplementary material for: Flotation Restricted Environmental Stimulation Therapy for Chronic Pain: A Randomized Clinical Trial
Source: JAMA Netw Open. 2021 May 14;4(5):e219627. doi: 10.1001/jamanetworkopen.2021.9627 (PMC8122226; doi:10.1001/jamanetworkopen.2021.9627)
Supplement: Supplement 1. — Trial Protocol and Statistical Analysis Plan [file jamanetwopen-e219627-s001.pdf]

This supplement contains the following items:

1. Original study protocol (the version number 1.1 follows internal nummeration. This protocol was approved by the local ethics committee prior to study begin and was not altered thereafter)
2. Original statistical analysis plan (included within the study protocol)

Floating for Chronic Pain

(Float4Pain)

Study Protocol

Version 1.1

23rd January 2018

Contents

Contents 2

Responsibilities 3

Rationale and Objectives 3

Study Design 4

Study Category 4

Endpoints 4

Study assessments 5

Study population / Sample size calculation 5

Inclusion criteria 6

Exclusion criteria 6

Study procedures on each study day 6

Dietary/Lifestyle restrictions and concomitant medication 7

Data handling 7

Risks 7

Floating 7

Data analysis 8

Amendments 8

Publication clause 9

Bibliography 9

Attachments 10

Responsibilities

Univ.-Prof. Dr. phil. nat. Florian Beißner
Somatosensory and Autonomic Therapy Research
Institute for Neuroradiology
Hannover Medical School
Carl-Neuberg-Str. 1, 30625 Hannover
Tel.: +49 511 5350 8413
Beissner.Florian@mh-hannover.de

Date, Signature

Prof. Dr. med. Matthias Karst
Pain Outpatient Department
Clinic for Anaesthesiology and Intensive Care Medicine
Hannover Medical School
Carl-Neuberg-Str. 1, 30625 Hannover
Tel.: +49 511 532 3108
Karst.Matthias@mh-hannover.de

Date, Signature

Jorge Manuel Sánchez, M. Sc.
Somatosensory and Autonomic Therapy Research
Institute for Neuroradiology
Hannover Medical School
Carl-Neuberg-Str. 1, 30625 Hannover
Tel.: +49 511 5350 8415
ManuelSanchez.Jorge@mh-hannover.de

Date, Signature

This study is a collaboration of the Institute for Neuroradiology and the Clinic for Anaesthesiology and Intensive Care Medicine.

Rationale and Objectives

In recent years there has been a novel approach for the treatment of chronic pain: Floatation REST (restricted environmental stimulation therapy). Floating is a therapeutic approach developed in the 1950s [6, 7] which patients float in a specialized pool or tank on water that contains high concentrations of Epsom salt (Magnesium sulfate). Water is kept at skin temperature (35-36ºC) and the pool or tank is shielded to provide almost complete darkness and silence. This therapeutic approach has proven to be effective in alleviating chronic pain [2].

While the high salinity of the water allows effortless floating and thus leads to muscle relaxation, the dark and silent environment without somatosensory stimulation greatly reduces sensory input. Thus, the two main mechanisms through which floatation REST may influence pain are the floatation itself and the sensory deprivation.

Due to the difficulties associated with designing a credible placebo control there have been no randomized controlled trials (RCTs) with patients blinding so far. Such blinding, however, is crucial, as the futuristic design of the pools / tanks and the unusual form of intervention are prone to elicit large placebo effects.

Here, we plan to conduct the first patient blinded RCT with a credible placebo control for floating and a no-treatment group.

Study Design

To investigate the therapeutic effects of floating and sensory deprivation on chronic pain we will divide the participants into three groups using a central randomization procedure.

- The **intervention group** will have the full floating experience: high buoyancy and maximal sensory deprivation.
- The **control group** will have a minimal floating experience designed as a placebo control. To achieve this we will manipulate the two mechanisms mentioned above: the water will have reduced buoyancy (low salinity) and there will be visual, auditory and somatosensory stimuli to reduce sensory deprivation to a minimum.
- The **no-treatment group** will be a waiting-list control. Patients in this group will not receive any treatment.

Our study will investigate short- and long-term effects of floating by evaluating separate endpoints after each floating session and after completion of the study (i.e. after five floating sessions).

Study Category

This study is a monocentric, patient-blinded, randomised and placebo-controlled study.

Endpoints

The study has **short-term endpoints** to assess the effect of each single floating session and **long-term endpoints** to assess the effect of a course of five successful floating sessions. While the former will be assessed before and after each floating session, the latter will be assessed one week after the final floating session and in two follow-ups at 12 and 24 weeks after completion. The only exception are pain area and widespreadness that will not be included in the follow-up assessments.

The study is successful, when the primary outcome shows significant differences between the intervention group and the control group at first assessment (one week after the final floating session).

Primary outcome (long-term):

The primary endpoint of the study is the reduction of pain (maximum and average) as measured by a numeric rating scale (NRS) retrospectively for one week.

Secondary long-term outcomes comprise improvements in
1.) Reduction of pain (maximum and average) by 30% or more (dichotomous)
2.) Pain-related disability (PDI)
3.) Trait Anxiety (STAI Trait)
4.) Depression (BDI)
5.) Quality of life (SF-12)
6.) Quality of sleep (NRS)
7.) Use of pain medication

Secondary short-term outcomes comprise improvements in
1.) Acute pain intensity (NRS)
2.) Acute pain area and widespreadness (from electronic pain drawing)
3.) State Anxiety (STAI State)
4.) Heart rate variability (improvement of sympatho-vagal balance indicated by an increase in HF- and a decrease in LF-HRV)
5.) Unusual bodily sensations during floating (electronic drawing)
6.) Altered states of consciousness during floating (5D-ASC)

Study assessments

1.) All pain scores will be acquired using numeric rating scales from 0 (“no pain”) to 100 (“maximum imaginable pain”). Ratings will assess the period of one week before the day of assessment.
2.) Pain-related disability, state and trait anxiety, depression and quality of life will be assessed using the following validated questionnaires
    a.) Pain disability index, PDI [9]
    b.) State-Trait Anxiety Inventory, STAI [10]
    c.) Beck Depression Inventory, BDI-II [1]
    d.) 12-Item Short Form Survey, SF-12 [11]

    All ratings will assess the period of one week before the day of assessment.
3.) Pain area and widespreadness (Widespread pain index, WPI) will be derived from electronic pain drawings acquired using the tablet PC application “SymptomMapper” developed by our group. Ratings will assess the period of four weeks before the day of assessment.
4.) HRV will be measured before and after each floating session using a Nilas MV Diagnostik- und Regulationssystem (Nilas MV GmbH, Hamburg).
5.) Unusual bodily sensations during floating will be acquired from electronic drawings with the “SymptomMapper” software.
6.) Altered states of consciousness will be assessed using the 5-Dimensional Altered States of Consciousness Rating Scale, 5D-ASC [4].
7.) Sleep quality will be assessed with a numeric rating scale. Patients will be asked how much their pain impairs their sleep on a scale from 0 (“not at all”) to 100 (“very much”).
8.) Use of pain medication will be assessed by patients’ self report.
9.) To assess the treatment belief, subjects will receive the following written question ‘‘Which treatment do you believe you received?’’ and offered the replies ‘‘real floating’’, ‘‘placebo floating’’ or ‘‘uncertain’’.

10.) To assess credibility of and expectancy in the treatment, a four-item Borkovec and Nau's self-assessment scale will be used [3].

Study population / Sample size calculation

Sample size calculation is based on a previous explorative studie [5] that showed significant differences between a treatment and a no-treatment group:

	Treatment (20)	No-treatment (17)
Pain before	61.75 ± 18.78	59.06 ± 17.68
Pain after	37.25 ± 30.22	60.41 ± 17.21

With this number we compute the difference post-pre, which yields -24.50 ± 35.58 for the treatment group and 1.35 ± 24.67 for the no-treatment group. These differences give a Cohen's d-value of 0.83.

For a two-tailed, two-sample t-test, it means that we need a sample size of 24 subjects per group (α=0.05, 1-β=0.95, allocation ratio 1:1). To account for attrition we will recruit 25 patients per group, i.e. 75 in total. Drop-outs will be replaced should the size of any group drop below 24 patients.

Groups will be balanced for age, gender and average pain intensity (retrospective NRS rating of the last week), and subjects will be recruited according to the following criteria.

Inclusion criteria

- Male and female patients, aged 18-75 years.
- Women will be considered for inclusion if they are not pregnant (self report) and not nursing.
- Current diagnosis of chronic pain disorder with somatic and psychological factors (ICD F45.41).
- Able and willing to give written informed consent.

Exclusion criteria

- Previous experience with floating.
- Acute illnesses that may exacerbate or shadow the chronic pain condition (acute inflammation, major depression, active neoplasm, etc.)
- History of alcohol or drug abuse.
- Any clinically relevant abnormal findings in the clinical history, which, in the opinion of the investigator may either put the subject at risk because of participation in the study, or may influence the results of the study, or the subject's ability to participate in the study.
- Any clinical condition that may prevent the subject from floating, such as: open injuries, contagious diseases (influenza, GI infections, athlete's foot), GERD, epilepsy, claustrophobia, schizophrenia, incontinence or acute skin disorders.
- Suspected inability to understand the protocol requirements, instructions and study-related restrictions, the nature, scope, and possible consequences of the study.

Study procedures on each study day

The subject recruitment will start in February 2018 according to the inclusion and exclusion criteria and will be conducted at the Pain Outpatient Department (Schmerzambulanz) of the MHH.

At visit 1 (screening), written informed consent will be obtained. Subjects will undergo a physical examination and medical history taking. In- and exclusion criteria will be checked. Subjects will be familiarized with the floating tank. After this, the primary and secondary long-term outcomes will be assessed, as well as the expectancy/credibility rating.

At visits 2 - 6 (intervention), each separated by 4 ± 2 days, in- and exclusion criteria will be checked again. Secondary short-term outcomes 1, 2 and 3 will be assessed. After this, a 5-minute ECG will be recorded for HRV analysis (short-term outcome 4). Subjects will then put on their bathing suits, take a shower and insert ear plugs before entering the floating tank (Tranquility, Floataway, Hingham, GB). The float itself will take 90 minutes. After the floating session subjects will leave the tank and take a shower to remove excess saline. Another 5-minute ECG will be recorded for HRV analysis (short-term outcome 4), before short-term outcomes 5 and 6 will be assessed.

After the last intervention, the subject is provided with a sealed envelope containing the questionnaires for the assessment of primary and secondary long-term outcomes and discharged.

On day 7 after visit no. 6 the subject is called by telephone and asked to fill out all questionnaires in the envelope and return it by mail.

This will be repeated at 12 weeks and 24 weeks after visit no. 6. Subjects in the intervention and control group will be unblinded after receipt of the last questionnaires.

Dietary/Lifestyle restrictions and concomitant medication

Subjects must not drink coffee for 6 hours and alcohol for 24 hours before each floating session.

Subjects should not shave any part of their body for 6 hours before each floating session.

The use of prescribed medications for the pain condition as well as acute medications that do not interfere with the study, as judged by the investigator, is permitted during the study, if necessary.

Subjects must continue their prescribed medication during the intervention and must not start any new therapeutic intervention (pharmacological or other).

The administration of acute and prescribed medication will be documented giving the name of the drug, the applied dosage, the time-point, and the reason.

Data handling

All source data will be saved pseudonymously. There will be a reference list, which will be kept separated from the study data. All responsible persons are bound to maintain confidentiality. Source data will be kept for at least ten years. Subjects are entitled to ask for the immediate deletion of their data without any prejudices.

Risks

Specific risks associated with study-related procedures are described below. Measures taken to minimize risks are included within each section. Overall, the scientific benefit that this research project delivers outbalances the minor risks that the study procedures might put on individual subjects.

Floating

Floating is a procedure employed for relaxation and stress reduction in many wellness centres around the world. It is generally considered very safe [8]. The water depth is around 25 cm and there is no risk of drowning even during sleep, since the high buoyancy of the saline allows effortless floating. In the control group (with lower salinity), water depth will be held around 10 cm.

Epsom-salt is non-toxic. Contact with the salty water can cause irritation of the eyes, mouth, nose and open wounds. Therefore, we will provide Vaseline to cover small scratches before floating. Inside of the tank there will be a damp cloth easily reachable for the subject.

The tank is equipped with an electric opening mechanism that can be operated from the outside and by the person inside the tank. The system is battery powered but the lid can still be opened in case of a system failure. Subjects can activate the light inside the tank or call the investigator via intercom by pressing one of three large buttons that are easily reachable.

Air circulation in the tank is ensured by thermal convection through an opening close to the face region and does not require any active components.

Water quality is assured by several factors: Subjects have to shower before entering the tank. The high salt levels of the water reduce bacterial growth to a minimum. The entire water of the tank is filtered automatically several times after each session and at the same time disinfected by an in-build hydrogen-peroxide/UV light sterilization unit. Water quality is monitored regularly by the principal investigator and water/salt will be changed after 200 floats.

Susceptible subjects may experience nausea, vertigo or disorientation in the tank that should, however, resolve spontaneously or by switching on the light inside the tank.

Data analysis

All statistical analyses will be conducted using R (https://www.r-project.org/) and Python (https://www.python.org/). Pain and sensation drawings will be analysed with the FMRIB Software Library (https://fsl.fmrib.ox.ac.uk/fsl/).

The statistical test for the primary outcome will be a two-tailed two-sample t-test of the differences (before treatment - one week after treatment). In case of significant differences in the primary outcome measure at baseline, an ANCOVA model will be used instead. If the prerequisites for t-tests are not granted, we will use non-parametric tests to compare the differences. The study will be positive if we observe a significant difference ($p<0.05$) between the intervention and the control group.

For the secondary long-term outcomes we will use Chi-squared test (for the dichotomous condition) and mixed ANOVAs with between-subject factor “group” and within-subject factor “time” for the other outcomes. Secondary short-term outcomes will be assessed using two-tailed paired t-tests.

All outcomes will be assessed in an Intention-to-Treat (ITT) analysis under the following conditions:

- The subjects who drop out before the first session will be excluded from the analysis and replaced by other subjects.
- We will continue to acquire the endpoints, keeping the same time intervals, i.e. 1, 12 and 24 weeks after the last session.
- In the case that we cannot assess the endpoint due to missing data, the subject will be excluded from the analysis and replaced by another subject.

In order to test for the robustness of the therapeutic effect, we will also conduct per-protocol analyses afterwards.

Amendments

All amendments to this protocol have to be presented to the ethical committee of the Hannover Medical School.

Publication clause

The data will be treated according to the privacy statement signed by the subjects. This privacy agreements states that the data will be stored pseudonymously for at least ten years by the responsible persons. Only the responsible people will have access to the reference list. Furthermore all responsible persons are bound to maintain confidentiality. The data might be published or given to third parties pseudonymously for scientific purposes.

The subjects will be able to enquire about the data and they are entitled to ask for the deletion of it without prejudices. The only data which cannot be deleted is their agreement for the participation in the study.

Bibliography

[1] Beck, A. T., Steer, R. A. and Brown, G. K., *Beck depression inventory-II*, San Antonio **78**, 490–498 (1996).

[2] Bood, S., Sundequist, U., Kjellgren, A., Nordstrom, G. and Norlander, T., *Effects of floatation-restricted environmental stimulation technique on stress-related muscle pain: what makes the difference in therapy - attention-placebo or the relaxation response?*, Pain Research and Management **10**, 201–209 (2005).

[3] Borkovec, T. D. and Nau, S. D., Credibility of analogue therapy rationales, Journal of Behavior Therapy and Experimental Psychiatry **3**, 257–260 (1972).

[4] Dittrich, A, Lamparter, D and Maurer, M, 5D-ABZ: *Fragebogen zur Erfassung Aussergewöhnlicher Bewusstseinszustände - Eine kurze Einführung*, PSIN Plus, Zürich (1999).

[5] Kjellgren, A., Sundequist, U., Norlander, T. and Archer, T., *Effects of flotation-REST on muscle tension pain, Pain Research and Management* **6**, 181–189 (2001).

[6] Lilly, J. C. *Effects of Physical Restraint and of Reduction of Ordinary Levels of Physical Stimuli on Intact Healthy Persons*, In: Meetings of the Group for the Advancement of Psychiatry. 1955, pp. 1–13.

[7] Lilly, J. C., *The Deep Self - Profound Relaxation and the Tank Isolation Technique*, 1st ed., (Simon and Schuster, 1977).

[8] Norlander, T., Kjellgren, A. and Bood, S. Åke. *Rehabilitation through flotation-REST (Restricted Environmental Stimulation Technique)*. In: Float Summit. 2012.

[9] Pollard, C. A., *Preliminary Validity Study of the Pain Disability Index , Perceptual and Motor Skills* **59**, 974–974 (1984).

[10] Spielberger, C. D. and Sydeman, S. J. *State-Trait Anxiety Inventory and State-Trait Anger Expression Inventory*, In: Lawrence Erlbaum Associates, Inc, 1994, pp. 292–321.

[11] Ware, J. J., Kosinski, M. and Keller, S. D., *A 12-Item Short-Form Health Survey: construction of scales and preliminary tests of reliability and validity*, Medical care **34**, 220–233 (1996).

Attachments

- CE certificate Nilas HRV device
- Technical information for Floating Tank
