# Supplementary material for: Flotation Restricted Environmental Stimulation Therapy for Chronic Pain: A Randomized Clinical Trial
Source: JAMA Netw Open. 2021 May 14;4(5):e219627. doi: 10.1001/jamanetworkopen.2021.9627 (PMC8122226; doi:10.1001/jamanetworkopen.2021.9627)
Supplement: Supplement 2. — eFigure. Short-term Differences (Postintervention Minus Preintervention) for Each Session eTable 1. Measurements During Placebo Floating eTable 2. Time of the Year When the Different Groups Floated eTable 3. Dropout Reasons eTable 4. Unusual Sensations During Flotation-REST and Placebo Floating eTable 5. Normalized Heart Rate Variability Measures [file jamanetwopen-e219627-s002.pdf]

# Supplementary Online Content

Loose LF, Manuel J, Karst M, Schmidt LK, Beissner F. Flotation restricted environmental stimulation therapy for chronic pain: a randomized clinical trial. *JAMA Netw Open*. 2021;4(5):e219627. doi:10.1001/jamanetworkopen.2021.9627

**eFigure.** Short-term Differences (Postintervention Minus Preintervention) for Each Session
**eTable 1.** Measurements During Placebo Floating
**eTable 2.** Time of the Year When the Different Groups Floated
**eTable 3.** Dropout Reasons
**eTable 4.** Unusual Sensations During Flotation-REST and Placebo Floating
**eTable 5.** Normalized Heart Rate Variability Measures

This supplementary material has been provided by the authors to give readers additional information about their work.

© 2021 Loose LF et al. *JAMA Network Open*.

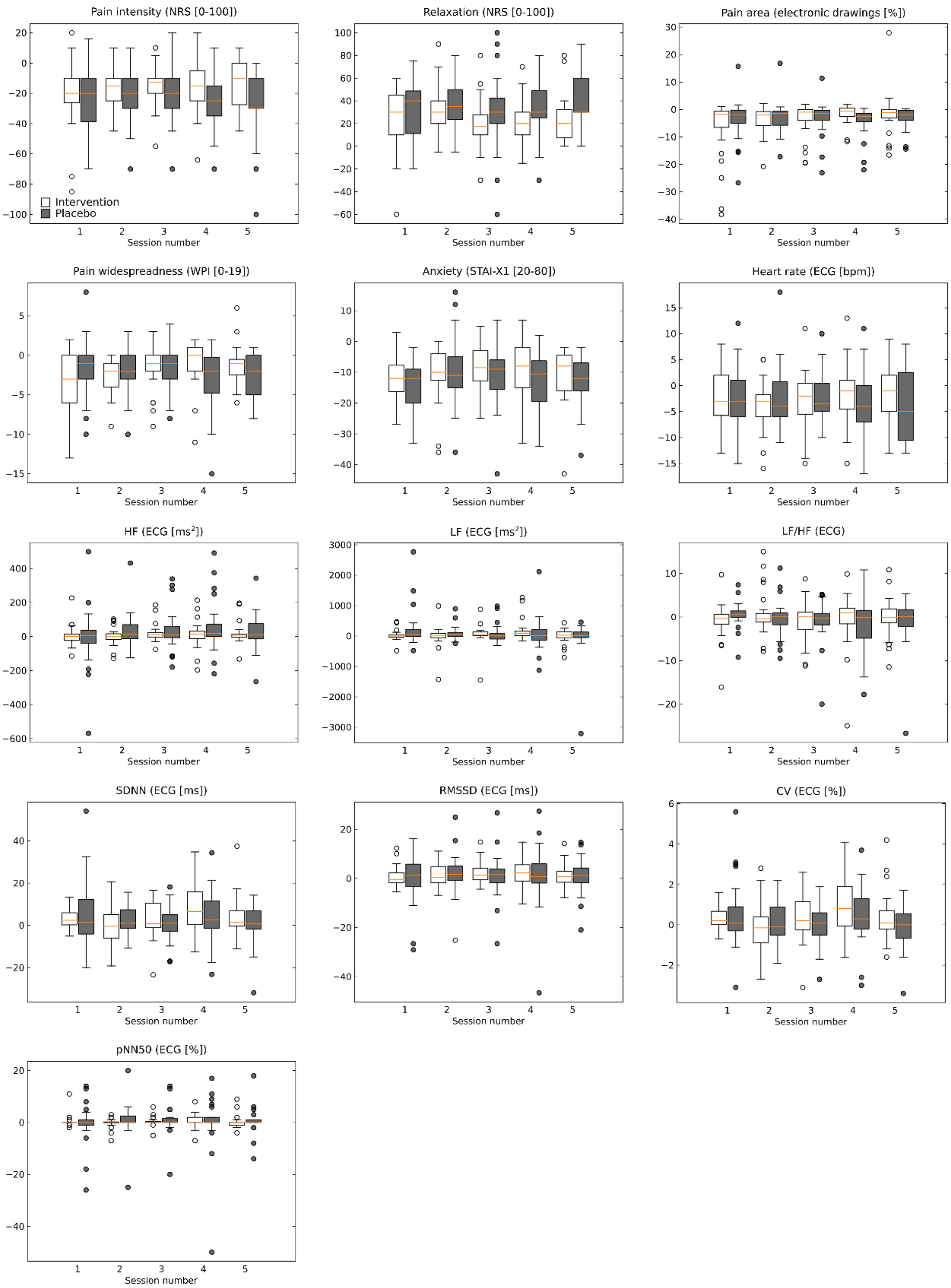


**eFigure. Short-term Differences (Postintervention Minus Preintervention) for Each Session** The intervention group is shown in white, and the placebo group in gray.

© 2021 Loose LF et al. *JAMA Network Open*.

**eTable 1. Measurements During Placebo Floating** To prevent a relaxation response, we attached a blood pressure cuff to the lower leg and measured every 4.0±0.7 minutes with closed lid. Furthermore, we asked every 10.3±2.4 minutes about their actual pain and relaxation. Abbreviations: BP, blood pressure; Q, questions.

| Minute | Measure | Minute | Measure | Minute | Measure |
|---|---|---|---|---|---|
| 1 | BP | 31 | Q | 63 | Q |
| 2 | Q | 33 | BP | 64 | BP |
| 5 | BP | 36 | BP | 68 | BP |
| 8 | BP | 40 | BP | 71 | Q |
| 10 | Q | 44 | Q | 72 | BP |
| 12 | BP | 45 | BP | 77 | BP |
| 17 | BP | 48 | BP | 80 | BP |
| 21 | BP | 52 | BP | 84 | Q |
| 23 | Q | 53 | Q | 85 | BP |
| 24 | BP | 57 | BP | 89 | BP |
| 29 | BP | 61 | BP | | |

© 2021 Loose LF et al. *JAMA Network Open*.

**eTable 2. Time of the Year When the Different Groups Floated** We were forced to cluster groups of the same group together as we could not change the salt concentration after every float.

| Time interval | Group | # subjects | # dropouts |
|---|---|---|---|
| 2018-06-26 - 2018-11-13 | Intervention | 4 | 0 |
| 2019-01-21 - 2019-04-06 | Placebo | 14 | 1 |
| 2019-04-29 - 2019-07-20 | Intervention | 16 | 2 |
| 2019-08-05 - 2019-10-02 | Placebo | 14 | 3 |
| 2019-10-07 - 2019-11-12 | Intervention | 7 | 1 |
| 2019-11-13 - 2019-12-02 | Placebo | 2 | 0 |
| 2019-12-03 - 2019-12-17 | Intervention | 1 | 0 |

© 2021 Loose LF et al. *JAMA Network Open*.

**eTable 3. Dropout Reasons** Subjects who dropped out prior to any measurement were not further analyzed. All other subjects were included in the analysis (pairwise deletion), as we did not find any systematic reason for dropping out of the study.

| Reason | Intervention | Placebo | Wait list |
|---|---|---|---|
| **Before baseline assessment** | | | |
| Loss of interest | 1 (3%) | 4 (11%) | 3 (10%) |
| Illness | 2 (6%) | 0 | 0 |
| Illness of a relative | 1 (3%) | 0 | 0 |
| Pregnancy | 0 | 1 (3%) | 0 |
| Enrollment in another study | 0 | 0 | 1 (3%) |
| New job | 0 | 1 (3%) | 0 |
| **After baseline assessment** | | | |
| Illness | 0 | 1 (3%) | 0 |
| Loss of interest | 0 | 0 | 1 (3%) |
| **After one session** | | | |
| Otitis | 1 (3%) | 0 | 0 |
| Herpes zoster | 0 | 1 (3%) | 0 |
| Too cold water | 0 | 1 (3%) | 0 |
| **After two sessions** | | | |
| Haemorrhoids | 1 (3%) | 0 | 0 |
| Gastroenteritis | 0 | 1 (3%) | 0 |
| **After three sessions** | | | |
| Illnes of a relative | 0 | 1 (3%) | 0 |
| **After four sessions** | | | |
| Loss of contact | 1 (3%) | 1 (3%) | 0 |

© 2021 Loose LF et al. *JAMA Network Open*.

**eTable 4. Unusual Sensations During Flotation-REST and Placebo Floating** Thirty-four participants (intervention: 18 (64%); placebo: 16 (52%)) experienced unusual bodily sensations during their sessions. Sensations were reported in 75 out of 265 sessions.

| Sensation | Intervention | Placebo |
|---|---|---|
| Cold | 5 (18%) | 5 (16%) |
| Warm | 3 (11%) | 5 (16%) |
| Tingling | 5 (18%) | 2 (6%) |
| Heavy | 6 (21%) | 1 (3%) |
| Hot | 3 (11%) | 3 (10%) |
| Radiating | 2 (7%) | 2 (6%) |
| Pain recedes | 1 (4%) | 2 (6%) |
| Burning | 1 (4%) | 2 (6%) |
| Swimming/diving in the sea | 1 (4%) | 1 (3%) |
| Light | 1 (4%) | 1 (3%) |
| Pressing | 2 (7%) | 0 |
| Dull | 2 (7%) | 0 |
| Being in the womb | 2 (7%) | 0 |
| Shooting | 1 (4%) | 0 |
| Tender | 1 (4%) | 0 |
| Pricking | 1 (4%) | 0 |
| Disembodied | 1 (4%) | 0 |
| Dead relatives appear | 1 (4%) | 0 |
| Lying on plasticine | 1 (4%) | 0 |
| Lying in a shell | 1 (4%) | 0 |
| Someone pressing the thumb | 1 (4%) | 0 |
| Spine unfolds | 1 (4%) | 0 |
| Water moving in the knee | 1 (4%) | 0 |
| Spinning in the water | 1 (4%) | 0 |
| Standing | 1 (4%) | 0 |
| Stinging | 0 | 1 (3%) |
| Blocked thoughts | 0 | 1 (3%) |
| Tangled dreams | 0 | 1 (3%) |

© 2021 Loose LF et al. *JAMA Network Open*.

**eTable 5. Normalized Heart Rate Variability Measures** This table supplements Table 3 by including total power, and normalized spectral measures (normalized to the total power). These measures showed neither differences between the groups nor pre-post differences. Abbreviations: LF - low frequencies (0.04-0.15 Hz); HF - high frequencies (0.15-0.4 Hz); TP - total power (0-0.4 Hz).

| | **Intervention** | | **Placebo** | |
|---|---|---|---|---|
| **Measure** | **pre** | **post** | **pre** | **post** |
| LF-power [$ms^2$] | $300.9 \pm 433.5$ | $351.0 \pm 456.1$ | $360.8 \pm 542.7$ | $433.5 \pm 644.1$ |
| HF-power [$ms^2$] | $74.7 \pm 104.7$ | $85.1 \pm 103.8$ | $99.3 \pm 103.2$ | $126.0 \pm 155.4$ |
| Total power [$ms^2$] | $753.3 \pm 832.5$ | $947.2 \pm 924.9$ | $863.6 \pm 843.3$ | $1057.5 \pm 1218.6$ |
| LF/TP [%] | $34.0 \pm 17.2$ | $33.1 \pm 15.8$ | $34.2 \pm 15.5$ | $34.6 \pm 13.7$ |
| HF/TP [%] | $11.5 \pm 10.9$ | $10.7 \pm 8.9$ | $12.7 \pm 10.6$ | $12.7 \pm 11.3$ |
| LF/HF | $6.0 \pm 6.1$ | $5.7 \pm 5.4$ | $5.5 \pm 5.9$ | $4.9 \pm 4.0$ |

© 2021 Loose LF et al. *JAMA Network Open*.
