# Supplementary material for: Flotation Restricted Environmental Stimulation Therapy for Chronic Pain: A Randomized Clinical Trial
Source: JAMA Netw Open. 2021 May 14;4(5):e219627. doi: 10.1001/jamanetworkopen.2021.9627 (PMC8122226; doi:10.1001/jamanetworkopen.2021.9627)
Supplement: Supplement 3. — Data Sharing Statement [file jamanetwopen-e219627-s003.pdf]

Data Sharing Statement

Loose. Flotation Restricted Environmental Stimulation Therapy for Chronic Pain. *JAMA Netw Open*. Published May 14, 2021. doi:10.1001/jamanetworkopen.2021.9627

Data
Data available: Yes
Data types: Deidentified participant data
How to access data: Raw data as well as necessary scripts for reproducing the results of this study are available at https://zenodo.org/record/4686867 (DOI: 10.5281/zenodo.4686867).
When available: April 14, 2021

Supporting Documents
Document types: Statistical/analytic code
How to access documents: Raw data as well as necessary scripts for reproducing the results of this study are available at https://zenodo.org/record/4686867 (DOI: 10.5281/zenodo.4686867).
When available: April 14, 2021

Additional Information
Who can access the data: Anyone
Types of analyses: For any purpose
Mechanisms of data availability: Open access repository
